# Editorial: Stress Induced Neural Changes in Emotional Disorders

**DOI:** 10.3389/fpsyt.2021.710691

**Published:** 2021-06-24

**Authors:** Fushun Wang, Fang Pan, Yiyuan Tang, Jason H. Huang

**Affiliations:** ^1^Institute of Brain and Psychological Science, Sichuan Normal University, Chengdu, China; ^2^Department of Medical Psychology, Shandong University Medical School, Jinan, China; ^3^Department of Psychological Sciences, Texas Technological University, Lubbock, TX, United States; ^4^Department of Neurosurgery, Baylor Scott & White Health, Temple, TX, United States; ^5^Department of Surgery, Texas A&M University College of Medicine, Temple, TX, United States

**Keywords:** emotional disorders, depression, neuromodulator, HPA axis, stress

Psychological processes include two equally important aspects of mental processes: cognition and emotion, emotional disorders might account for more than 90% of mental disorders. For example, major depression is a prevalent emotional disorder that affects more than 1/5 of the populations worldwide, making it one of the most prevalent health-related causes of human suffering. Moreover, it is a leading risk factor for the estimated one million deaths by suicide per year world-wide. Despite that emotion is critically important to us, emotion is one of the least studied biological phenomena of the brain, the mechanism of the emotional disorders is not clear. Thus, the treatment is not as effective as expected, which might be effective in only a subset of patients and acts slowly (1).

Stress has been regarded as a critical causing factor for emotional disorders. Stress is an evolutionarily adaptive response to deal with situations that impact threat to the organism and require rapid “flight or fight” responses (2). Stress is evolutionarily important for survival and benefits to all lives, however, overwhelming stress is considered to one of the main risk factors for the development of many emotional disorders such and anxiety, depression. For example, the onset of major depression are often correlated with stressful events in earlier lives, many studies reported significant correlation between the onset of major depression and the number of traumatic events within 3 months before the onset of the disease. In addition, stress can happen very long ago, for example, early life stress can induce emotional depression in adult lives (3). This means stress can induce long term changes in the body to induce emotional disorders.

The stress induced neural changes are very complicated. There is compelling evidence of a causal link between chronic stress and the sympathetic system as well as the HPA axis (hypothalamus-pituitary-adrenal axis) and emotional disorders. Stress causes elevation of corticosteroids and other stress hormones, such as CRF (corticotropic-releasing factor), the known stress hormone. HPA dysregulation may be both a causative factor and a consequence of emotional disorders. In addition to hypothalamus, the locus Coerleus (central norepinephrine) also plays an important role in stress. The major function of norepinephrine (NE) system has been known as “fight or flight,” or “fear or anger” emotions (4). As we reported before, everything around us happens in an anticipated way (expected) or not anticipated way (surprising). If it happens as expected, people feel calm; if it happens unexpected, people will feel scared or angry (5). Therefore, chronic unpredictable stresses are most often used in animal models of depression. When the animals are faced with stress, they often respond with fear or anger emotions, and “fight or flight” responses trying to get rid of these stressors. After a long term of failure to deal with these stressors, they learned the “helplessness” and try to live with these stressors sadly. We say that these animals are depressed and the depression model is set up.

Furthermore, stress can induce long term changes in the endocrine systems, which in turn induce behavior changes that habitually deal with the stressful situations, thus cytokine is also regarded as a causing mechanism for depression. Lymphocytes, mast cells and other immune system cells contain adrenoreceptors that respond to the peripheral release of NE. The central NE system innervates many vital parts of the immune system- the lymph organs, spleen, thymus. It is reported that lymphocyte activity was dramatically reduced in the depressed patients. There is evidence that sympathetic nervous, immune and endocrine systems are linked. Moreover, some epigenetic changes or neural changes induced by early life stress might be involved in the emotional disorders. The expression of all receptors for previous neuromodulators, neurotransmitters, cytokines can be affected by epigenetics. These changes have been suggested to be involved in the emotional diseases.

In 2017, we proposed one topic “Stress Induced Neuroplasticity and Mental Disorders” for Neural Plasticity and got 41 submissions. In 2018, we proposed one topic titled “Early life stress and depression” for Frontiers in Psychiatry and got 42 submissions. This is a fast developing topic, therefore, here we continue to propose a similar topic to collect recent studies about the mechanisms of stress inducing emotional disorders, such as phobia, depression and anxiety. In this special issue, we received 27 submissions and got 18 papers accepted.

In the review paper, titled “The Locus Coeruleus- Norepinephrine System in Arousal: Unraveling Historical, Current and Future Perspectives,” the authors Ross and Van Bockstaele from Drexel University gave a detailed review about the arousal function of locus-coeruleus (LC)-NE system. They discussed technological advancements that chronologically led to our current understanding of the arousal system. The paper was reviewed by professors Leszek Kubin from University of Pennsylvania, and Shahzad Khan from Stanford University, who gave a high praise for this paper: “A strength of this review is its detailed and thorough overview of stress and arousal research.” “To cover this broad topic based on several subfields of neuroscience that, traditionally, developed along their own separate paths is an ambitious undertaking. The authors have largely succeeded in producing a text that has superior educational value.”

In an experimental paper, author Yang et al. from Zhejiang University, one of most famous universities in China, identified some specific genetic targets for the diagnosis and treatment of depression. They screened 325 differentially expressed genes, with 42 genes down-regulated, while 283 genes up-regulated in hippocampal tissue. The results showed a significant change in Wfs1 after chronic stress stimulation, and they suggested that Wfs1 can be used as a prognosis and treatment target for depression. The paper is titled “Wfs1 and Related Molecules as crucial Biomarkers in the Hippocampus of Depression.”

In the experimental paper, titled “Childhood maltreatment and depression in adulthood in Chinese female college students: the mediating effect of coping style,” Zheng et al. investigated the mediating effect of coping style in depression in adulthood among Chinese female college students. They used self-report questionnaires assessing childhood maltreatment, depression and coping style among 738 participants, and the results illustrated that childhood maltreatment could influence adult depression through the mediating role of coping style.

In the review paper titled “Autophagy-based hypothesis on the role of brain catecholamine response during stress,” the authors Limanaqi et al. investigated stress induced changes in the homeostatic balance of the catecholamine brain systems, including norepinephrine (NE)- and dopamine (DA)-containing neurons within the locus coeruleus (LC) and ventral tegmental area (VTA), which are readily and similarly activated by psychostimulants. They found that brain catecholamine response to stress results in time-dependent regulatory processes involving mesocorticolimbic circuits and networks, where LC-NE neurons respond more readily than VTA-DA neurons. In this mini-review, they also discussed the role of autophagy in brain catecholamine response to stress and those factors which may lead to their dysregulation. They suggested that autophagy plays a crucial role in the response and adaptation of LC-NE and VTA-DA systems to stress.

In the experimental paper “Electroacupuncture alleviates cerebral ischemia-reperfusion injury in rats by histone H4 lysine 16 acetylation-mediated autophagy,” the authors Xu et al. used acupuncture to treat animals, and found that acupuncture can change the autophagy via the reduction in lysine 16 of histone H4 acetylation. Their finding offer some epigenetic suggestion for stress induced depression, that regulating histone H4 lysine 16 acetylation-mediated autophagy may be a key mechanism for stress induced depression.

Maternal separation (MS) has been commonly used as a rodent model to identify the developmental effects of child neglect. In the paper “Early life neglect alters emotional and cognitive behavior in a sex-dependent manner and reduces glutamatergic neuronal excitability in the prefrontal cortex,” Sun et al. examined the impact of early-life stress on glutamatergic neuronal excitability in the prefrontal cortex. Rats were separated from the mom for 4 h per day on postnatal days 2–5 and for 8 h per day on postnatal days 6–16 and then weaned on day 17. The results showed that maternal separation reduced the number of glutamatergic neuron activities in male rats, and induced anxiety-like behavior, a passive coping strategy and increased fear memory in male rats and decreased locomotor activity in both sexes. In addition, maternal separation also slightly impaired working memory during non-stressful situations in female rats but did not change spatial reference memory or associative learning under stressful circumstances in either sex.

In another paper, which was titled “The effect of early maternal separation combined with adolescence chronic unpredictable mild stress on behavior and synaptic plasticity in adult female rat,” the authors Huang et al. evaluated the depression model of early MS combined with CUMS in female adult SD rats, and measured synaptophysin (SYN), postsynaptic density-95 (PSD-95) and growth-associated protein-43 (GAP-43) expressions in hippocampus were detected by western blot. The results showed that rats in MS+CUMS group presented more serious depression-like and anxiety-like behavior than in MS group. In addition, few Nissl bodies in the hippocampus CA1 and DG regions, less percentage of SYN-positive cells and down-regulated expressions of SYN, PSD-95, and GAP43 were found in hippocampus of rats in MS+CUMS group. They concluded that adult female rats that had undergone MS and CUMS performed more critical depression-like and anxiety-like behaviors, and this process may be resulted from synaptic plasticity impairing.

An experimental animal study, which was titled as “The differential role of cytokines on stress responses in ovariectomized female rats,” investigated the mechanisms for menopause induced anxiety and depression. The authors Park et al. applied stress to ovariectomized rats, and found that ovariectomy and stress combine to increase the depressive like behaviors and neuro-inflammatory responses. In addition, they show neuroinflammation as a potential contributor to depressive like symptoms during menopausal transition.

In the experimental paper, Zhang et al. studied another kind of sex related depression, premenstrual dysphoric disorder, which is a common mental health disturbance associated with several periodic psychological symptoms in women. The paper was titled “Paeonol at Certain Doses Alleviates Aggressive and Anxiety-like Behaviors in Two Premenstrual Dysphoric Disorder Rat Models.” Selective serotonin reuptake inhibitors (SSRIs) are the first-line treatment for PMS/PMDD patients; however, side effects are inevitable, especially in long-term treatment. In this study, they used a kind of natural compound paeonol, and found it to be effective in central nervous system disorders with its anti-inflammatory, anti-oxidant, and neuroprotective effects.

In the review paper, “Gender differences in depression: evidence from genetics,” Zhao et al. compared gender difference for depression in aspect of behavioral genetics. They found that gender differences exist in heritability and the gene associated with depression after reviewing relevant research. Both genes and gene-environment interactions contribute to the risk of depression in a gender-specific manner. They talked about the relationships between serotonin transporter gene-linked promoter region (5-HTTLPR) and depression in great detail.

The experimental paper, title “Perceived stress and life satisfaction among Chinese clinical nursing teachers: A moderated mediation model of burnout and emotion regulation,” investigated the underlying mechanisms of how and when perceived stress increased the risk of burnout and decreased life satisfaction among clinical teaching nurses. They used questionnaires about perceived stress, burnout, emotion regulation and life satisfaction were self-administered among 1,372 teaching nurses from eight tertiary military hospitals in China. The results revealed that perceived stress had direct and indirect impacts on life satisfaction, with the principal element of burnout- emotional exhaustion acting as a mediator. Moreover, the association between perceived stress and emotional exhaustion was moderated by emotion suppression- a key emotion regulation strategy.

In the interesting study, the authors Song et al. investigated the effects of a kind of Chinese herb on depression. The paper is titled “Effects of Xiaoyaosan on Depressive-Like Behaviors in Rats with Chronic Unpredictable Mild Stress through HPA Axis Induced Astrocytic Activities.” They investigated the behavioral changes of chronic unpredictable mild stress (CUMS), the expression of corticosterone of the HPA axis, the expression of Glutamate receptor and astrocytes glial fibrillary acidic protein in the hippocampus. The study proved that this kind of Chinese herb affects depression by affecting the HPA axis and glutamate recycling.

In another paper, “Effect of Jian-Pi-Zhi-Dong Decoction on the amino acid neurotransmitters in a rat model of Tourette Syndrome and Comorbid Anxiety Disorder,” the authors Zhang et al.studied another kind of Chinese herb with depression. They observed mitochondrial changes with transmission electron microscopy, and they also measured the content of Glutamate and γ-aminobutyric acid (GABA) both in the serum and striatum and the expression of their receptors by western blot and real-time polymerase chain reaction. The study revealed that the Chinese herb was effective in ameliorating the severity of behavioral symptoms of anxiety via increase in GABA levels and decreases in glutamate levels in the serum.

In one more paper, which was titled “Effect of the ZiBuPiYin recipe on diabetes-associated cognitive decline in Zucker diabetic fatty rats after chronic psychological stress,” Bi et al. examined the effect of another kind of Chinese herb ZiBuPiYin recipe on Zucker diabetic fatty rats and explored the impact of chronic stress on altered β-amyloid (Aβ) metabolism through insulin receptor substrate (IRS) 1/protein kinase B (AKT)/mammalian target of rapamycin (mTOR) signaling pathway after the induction of chronic psychological stress. They found that the herb treatment significantly decreased anxious-like behaviors and plasma corticosterone levels, and ameliorated learning and memory impairments of the rats after chronic psychological stress. So they suggested that the herb may be a potential therapeutic application for the treatment of depression induced by chronic psychological stress.

Inflammation has recently been found to be a causing factor for depression, in the experimental paper “Changes of serum melatonin, interleukin-6, homosysteine, complement C3 and C4 levels in patients with depression,” Tao et al. investigated the changes in serum concentrations of melatonin, interleukin-6 (IL-6), homocysteine, complement C3, and C4 in depression patients and relationships of them with depression activity. The results suggest that higher serum MT, IL-6, and hcy levels were correlated with pathogenesis of depression.

Similarly, in another paper, titled “Susceptibility to hyperglycemia in rats with stress- induced depressive-like behavior: involvement of IL-6 mediated glucose homeostasis signaling,” Li et al. also studied the role of inflammation in the pathogenesis of depression and insulin resistance. The results showed that CUMS exposure resulted in the depression-like behavior at various time points in rats. Moreover, the rats exhibited increased peripheral glucose levels, impaired hepatocytes and hippocampal neurons, and decreased hypothalamic GLUT4 levels after 6 week of CUMS exposure. The experiment also showed that 6-week CUMS stimulation also induced susceptibility to hyperglycemia, which is associated with IL-6-mediated inhibition of glucose homeostasis signaling in the hypothalamus.

In the paper “SGK1 (serum-and glucocorticoid-regulated kinase 1): into a straight line for the major depressive disorder,” Dattilo et al. reviewed the expression of both the brain-derived neurotrophic factor (BDNF) and the vascular endothelial growth factor (VEGF), and their effects in neurogenic activity. In addition, they reviewed studies about SGK1 on the expression of BDNF and VEGF, trying to depict SGK1 as molecular junction of the complex mechanisms underlying the MDD, in the effort to suggest the kinase as a potential biomarker and strategic target in a modern molecular antidepressant therapy.

In the experimental paper “Repeated nitrous oxide exposure exerts antidepressant-like effects through neuronal nitric oxide synthase activation in the medial prefrontal cortex,” the authors Liu et al. also investigated the neurogenesis effects of N_2_O on BDNF, and suggested that the antidepressant-like effects of N_2_O work through enhancing BDNF expression levels, which might underlie the pharmacological mechanism of the antidepressant-like effects of N_2_O exposure.

Taken together, several pathogenic hypotheses, such as monoamines deficiency and neurobiological alterations in the stress-responsive system, including the HPA axis, monoamine system and the immune system, have been proposed for MDD, recently, many studies point to neurogenesis (6). In this special issue, these 18 papers investigated stress induced depression-related behaviors, offering some new mechanisms, such as VEGF changes, BDNF changes, which might be modulated by N_2_O or SGK1. We hope these papers will shed some new light in the field of stress and emotional studies.

## Author Contributions

FW and JH did the writing. FP and YT helped with the revision. All authors contributed to the article and approved the submitted version.

## Conflict of Interest

The authors declare that the research was conducted in the absence of any commercial or financial relationships that could be construed as a potential conflict of interest.
